# Post-stroke fatigue: The role of comorbidities and its impact on quality of life

**DOI:** 10.1186/s12883-025-04143-2

**Published:** 2025-04-23

**Authors:** Marie Matérne, Mialinn Arvidsson Lindvall, Peter Appelros, Olivia Eriksson, Gustav Jarl

**Affiliations:** 1 School of Behavioural, Social and Legal Sciences, Örebro University, Örebro, Sweden; 2 School of Health, Care and Social Welfare, Mälardalen University, Västerås, Sweden; 3 University Health Care Research Center, Faculty of medicine and health, Örebro University, Örebro, Sweden; 4 Department of Prosthetics and Orthotics, Faculty of Medicine and Health, Örebro University, Örebro, Sweden

**Keywords:** Comorbidity, Post-stroke fatigue, Quality of life, Social factors, Stroke

## Abstract

**Background:**

Post-stroke fatigue (PSF) is a common complication following stroke that affects approximately 50% of stroke survivors.

**Purpose:**

The purpose of this study was to investigate the role of comorbidities in PSF and the impact of PSF on Quality of Life (QoL). To achieve this, residual stroke symptoms have also been considered.

**Methods:**

The participants were stroke survivors living in a Swedish municipality. Self-reported data were collected via the Fatigue Assessment Scale (FAS), the Riksstroke questionnaire, and the Short Form Health Survey 36 (SF-36). Linear multiple regression and Spearman’s correlation coefficient were used to analyze the data.

**Results:**

A total of 142 participants (83 men) with a mean age of 74.8 (SD 9.7) years were included in the study. Fatigue levels were classified as normal (FAS 10–21) for 70 (49.3%) individuals, mild-to-moderate (FAS 22–34) for 56 (39.4%) individuals, and severe (FAS 35–50) for 16 (11.3%) individuals. The mean FAS score was 23.3 (SD 8.2). Multiple regression analysis indicated that the presence of vertigo (β = 0.24, *p* = 0.004), chronic pulmonary disorders (β = 0.29, *p* = 0.003), and hemiparesis (β = 0.18, *p* = 0.05) were associated with more severe PSF. The model explained 19.2% of the variance in PSF. A higher level of PSF was associated with worse QoL in all eight SF-36 domains (*r* = -0.38 to -0.67).

**Conclusions:**

Vertigo, chronic pulmonary disorders, and hemiparesis were significantly associated with more severe PSF. Additionally, higher levels of fatigue were associated with a worse QoL. These findings confirm that PSF is a multifaceted phenomenon, underscoring the importance of addressing PSF in rehabilitation to improve outcomes for stroke survivors.

## Introduction

Stroke is one of the most common causes of disability and death worldwide [1] and may affect survivors not only physically [2] but also cognitively, psychologically [3, 4], and socially [5]. Approximately half of all stroke survivors experience post-stroke fatigue (PSF) [6]. PSF is characterized by a feeling of mental exhaustion that typically does not improve with rest. Many survivors report it to be their worst or among their worst stroke symptoms [7]. The consequences of PSF range across different life domains, and PSF may restrict the ability to perform work and participate in everyday life and leisure interests, which can lead to involuntary isolation [8].

Various factors may contribute to the development of PSF, such as pre-stroke affective disorders, cognitive impairment, and psychosocial and behavioral factors [7]. Comorbid diseases also play a role in the etiology of PSF [9], but it is unclear which comorbid conditions are most important in this respect.

Several studies have reported an association between PSF and reduced quality of life (QoL) among young stroke survivors (< 65 years) [10]. Compared with younger stroke survivors, older stroke survivors typically have more disabilities and comorbidities, but few studies have investigated the association between PSF and QoL in an unselected cohort of stroke survivors.

The purpose of this study was to investigate the role of comorbidities in PSF and the impact of PSF on QoL. To achieve this, residual stroke symptoms have also been considered.

## Materials and methods

All persons with stroke (*n* = 330) living in the municipality of Kumla, Sweden, the 31 th of December 2019 were eligible for this study [11]. These persons were identified through the Swedish stroke register, and of the medical record system of Örebro region. Their diagnosis was confirmed in the medical record by a physician [11]. The mean duration since the first stroke of this population was 8.3 years (0–37 years), 85% had ischemic stroke, 11% intracerebral hemorrhage and 4% subarachnoid hemorrhage [11].

### Procedures

An information letter describing the study, a consent form and the study questionnaires (see description below) were sent by post to the 330 persons identified living with stroke in Kumla. In total, 220 individuals agreed to participate in the study and returned the questionnaires. Among these participants, 142 answered the Fatigue Assessment Scale (FAS) and were thus included in this study.

### Measures

*The Fatigue Assessment Scale* (FAS) [12] measures fatigue with 10 questions answered on a five-point Likert scale, from Never to Always. The sum score ranges from 10 to 50, where 10–21 points indicate “normal” levels of fatigue, 22–34 points indicate mild-to-moderate fatigue, and 35–50 points indicate severe fatigue [13]. The Swedish version of FAS has been validated with stroke survivors and demonstrated strong internal consistency (α = 0.82) and good test–retest reliability (Intraclass Correlation Coefficient = 0.73) [12].

*The Riksstroke 1-year follow-up questionnaire* [14] is used for long-term follow-up of stroke patients by the Swedish Stroke Register (Riksstroke). It is validated for use in stroke survivors [15]. The questionnaire consists of 46 questions about the person’s living conditions, daily activities, support from health care and municipal care, rehabilitation, health condition and health interventions. A question regarding return to prestroke life and activities was included in the present study.

*The Short Form Health Survey 36 (SF- 36)* consists of 36 questions measuring an individual’s quality of life from 0–100 in eight domains; Physical functioning, Role—Physical, Bodily pain, General health, Vitality, Social functioning, Role—Emotional and Mental health [16]. The results on SF- 36 can further be calculated as two component scores, that is, the Physical component score (PCS) and Mental component score (MCS). The Swedish version of SF- 36 has demonstrated strong internal consistency (α > 0.80) for most socio-demographic subgroups [17].

*Data on comorbidities* were retrieved from medical records. The comorbidities were selected based on our previous publication [18], which included a comprehensive review of all disease groups. The conditions that had the greatest impact on general health status were included as covariates (predictors) in the current study. The assessment of each individual comorbidity was based on the severity of the findings described in the patient records. Most often, this was combined with the entry of a diagnosis code.

### Analysis

Two participants had one missing answer on the FAS (Items 4 and 9). These missing answers were imputed using the mean value of each participant’s answers on the remaining nine items. The mean and standard deviation (SD) for each item score and the sum score of the FAS, as well as linear regression analyses with the sum scores of the FAS as the dependent variable, were calculated.

First, we conducted a univariate regression analysis identifying variables with p values < 0.20. Second, these variables were investigated for collinearity by calculating Spearman’s correlation coefficient between all the variable pairs and calculating the variance inflation factor. We defined substantial collinearity as a correlation coefficient > 0.7 or < − 0.7 or a variance inflation factor > 2.5 [19]. Third, variables not demonstrating collinearity were entered into a multiple linear regression analysis via forward and backward commands in SPSS. Variables with *p* values < 0.05 in the multiple regression analysis were considered significantly associated with fatigue. Assumptions of the final regression model (homoscedasticity and normal distribution of residuals) were tested with the Breusch-Pagan and Shapiro-Wilks tests. Nagelkerke’s (unadjusted and adjusted) R^2^ was calculated as a measure of the proportion of variance the final model explained. We calculated the correlation between the FAS sum scores and the SF- 36 domains and between the FAS sum scores and return to pre-stroke life and activities. Spearman’s correlation coefficient was used, as the FAS sum scores were not normally distributed (Shapiro–Wilk test of normality, *p* < 0.001). Correlation coefficients of 0.00–0.10 were considered negligible, 0.10–0.39 weak, 0.40–0.69 moderate, 0.70–0.89 strong, and 0.90–1.00 very strong [20]. All analyses were conducted via IBM SPSS Statistics 29 for Windows (Armonk, NY: IBM Corp.).

### Ethics

The study was approved by the Swedish Ethical Review Authority (reference No. 2019–02359) and performed in accordance with the principles stated in the Declaration of Helsinki [21].

## Results

We identified the population through the Swedish stroke register, and of the medical record system of Örebro region as the 31 th of December 2019 and started the data collection in spring 2020 and finished about a year later. The study sample consisted of 142 participants who answered the FAS. Data on sex, age, and comorbidities were available for all participants. The Riksstroke questionnaire was completed by 139 participants, and 118 answered the SF- 36. The participants consisted of 83 (58.5%) men and 59 (41.5%) women with a mean age of 74.8 (SD 9.7) years. We compared the 142 participants to the 188 people in the dataset who did not answer the FAS (Table 1). When comparing the responders and non-responders, we found no significant differences for age (74.8 vs 74.3 years, *p* = 0.70), sex (58.5% vs 59.0% men, *p* = 0.91), time since first stroke (8.0 vs 8.6 years, *p* = 0.48) and stroke type (85.2% vs 85.1% ischemic stroke). We found two significant differences between responders and non-responders among comorbidities and residual stroke symptoms: responders had a lower prevalence of cognitive impairment (4.9% vs 17.6%, *p* < 0.001) and aphasia (6.3% vs 13.3%, *p* = 0.04).

**Table 1:** Comparisons of responders and non-responders

Demographic variables		Responders (*n* = 142)	Non-responders (*n* = 188)	*p* value ^a^
		N (%) or mean (SD)	N (%) or mean (SD)	
Sex, male		83 (58.5)	111 (59.0)	0.91
Age, years		74.8 (9.7)	74.3 (13.9)	0.70
Time since first stroke, years		8.0 (7.9)	8.6 (7.1)	0.48
Stroke type	Ischemic	121 (85.2)	160 (85.1)	0.98
	Hemorrhagic	21 (14.8)	28 (14.9)	
**Comorbidities and residual stroke symptoms**				
Diabetes mellitus		40 (28.2)	51 (27.1)	0.83
Orthopedic disorders		42 (29.6)	58 (30.9)	0.80
Vertigo		29 (20.4)	32 (17.0)	0.43
Ischemic heart disease		22 (15.5)	36 (19.1)	0.39
Cognitive impairment		7 (4.9)	33 (17.6)	< 0.001
Psychiatric disorders		12 (8.5)	23 (12.2)	0.27
Chronic pulmonary disorders		15 (10.6)	12 (6.4)	0.17
Hemiparesis		26 (18.3)	51 (27.1)	0.06
Aphasia		9 (6.3)	25 (13.3)	0.04

*SD* standard deviation

^a^ two-sided t-test was used for continuous variables and two-sided Chi-square test was used for categorical variables

The mean scores for individual FAS items ranged from 1.8 (median 2) (*I experience problems with thinking things through*) to 3.1 (median 3) (*I have enough energy to manage my everyday life*) (Table 2). The mean FAS sum score was 23.3 (SD 8.2), median 22. Fatigue levels were normal (10–21 points) for 70 (49.3%) individuals, mild–to-moderate (22–34 points) for 56 (39.4%) individuals, and severe (35–50 points) for 16 (11.3%) individuals.

**Table 2:** Findings of the Fatigue Assessment Scale (*n* = 142)

Item	Mean (SD)	Median (Q1; Q3)
1. I am troubled by fatigue	2.6 (1.2)	2 (2; 4)
2. I get tired very quickly	2.5 (1.2)	2 (2; 4)
3. I do a few things during the day	2.5 (1.2)	2 (2; 3)
4. I have enough energy to manage my everyday life ^a^	3.1 (1.2)	3 (2; 4)
5. I feel physically exhausted	2.2 (1.1)	2 (1; 2)
6. I find it difficult to make a start on things	2.2 (1.2)	2 (1; 2)
7. I experience problems with thinking things through	1.8 (1.0)	2 (1; 2)
8. I feel no desire to do anything	2.2 (1.1)	2 (1.8; 2)
9. I feel mentally exhausted	1.9 (1.1)	2 (1; 2)
10. I can concentrate quite well when I do something ^a^	2.3 (1.2)	2 (1; 3)
Total score	23.3 (8.2)	22 (17; 28)

The rating scale is scored as 1 = Never, 2 = Sometimes, 3 = Regularly, 4 = Often 5 = Always except for Items ^a^ 4 and 10 on which the scoring is reversed. Thus, higher scores indicate more severe fatigue on all items

*SD* standard deviation, *Q1* first quartile, *Q3* third quartile

In the univariate regression analysis, sex, vertigo, cognitive impairment, psychiatric disorders, chronic pulmonary disorders, and hemiparesis had *p* values < 0.20 (Table 3). As there was no substantial collinearity (*r* = − 0.07 to 0.25; the variance inflation factor was 1.0 to 1.1), all these variables were entered into the multiple regression. The results of the multiple regression analysis were the same when the forward and backward commands in SPSS were used. The Breusch-Pagan test did not reject homoscedasticity of the final model (*p* = 0.34). The Shapiro-Wilks test rejected normal distribution of the residuals (*p* < 0.001). Thus, we used bootstrapping to calculate standard errors and p-values in the final regression model. In this analysis, the presence of vertigo (β = 0.24, *p* = 0.004), chronic pulmonary disorders (β = 0.29, *p* = 0.003), and hemiparesis (β = 0.18, *p* = 0.05) were associated with more severe PSF. The final model explained 19.2% of the variance in the fatigue scores (unadjusted R^2^ = 19.2%, adjusted R^2^ = 17.5%).

**Table 3:** Results of regression analyses with Fatigue Assessment Scale (FAS) as the outcome (*n* = 142)

		**N (%) or mean (SD)**	**Mean FAS score (SD)**	**Median FAS score (Q1; Q3)**	**Univariate regression**			**Multiple regression**		
**Demographic variables (*n* = 142)**					**B (SE)**	**β**	***p* value**	**B (SE)**	**β**	***p* value**
Sex	Female	59 (41.5)	24.7 (8.6)	23 (18; 29)	2.3 (1.4)	0.14	0.10 ^a^			NS
	Male (ref)	83 (58.5)	22.4 (7.9)	20 (16; 27)						
Age, years		74.8 (9.7)	–-	–-	0.05 (0.07)	0.06	0.51			
**Comorbidities and residual stroke symptoms (*n* = 142)**										
Diabetes mellitus	Yes	40 (28.2)	22.9 (6.9)	23 (17; 27.8)	− 0.6 (1.5)	− 0.03	0.70			
	No (ref)	102 (71.8)	23.5 (8.7)	21 (17; 28)						
Orthopedic disorders	Yes	42 (29.6)	24.0 (7.6)	24 (18; 29)	1.0 (1.5)	0.06	0.52			
	No (ref)	100 (70.4)	23.0 (8.5)	20 (17; 27)						
Vertigo	Yes	29 (20.4)	28.0 (7.2)	27 (23.5; 34.5)	5.8 (1.6)	0.29	< 0.001 ^a^	4.9 (1.7)	0.24	0.004
	No (ref)	113 (79.6)	22.1 (8.1)	20 (16.8; 26)						
Ischemic heart disease	Yes	22 (15.5)	21.3 (7.4)	21.5 (13.8; 26.3)	− 2.4 (1.9)	− 0.10	0.22			
	No (ref)	120 (84.5)	23.7 (8.4)	22 (17; 29)						
Cognitive impairment	Yes	7 (4.9)	27.3 (9.6)	28 (16; 36)	4.2 (3.2)	0.11	0.19 ^a^			NS
	No (ref)	135 (95.1)	23.1 (8.1)	21 (17; 27)						
Psychiatric disorders	Yes	12 (8.5)	27.8 (9.9)	27.5 (20.8; 34.8)	4.9 (2.5)	0.17	0.047 ^a^			NS
	No (ref)	130 (91.5)	22.9 (8.0)	21 (17; 27)						
Chronic pulmonary disorders	Yes	15 (10.6)	30.3 (7.8)	28 (24; 35)	7.8 (2.2)	0.29	< 0.001 ^a^	7.7 (2.2)	0.29	0.003
	No (ref)	127 (89.4)	22.5 (7.9)	20 (17; 27)						
Hemiparesis	Yes	26 (18.3)	27.0 (9.5)	25 (20; 31.5)	4.5 (1.8)	0.21	0.012 ^a^	3.9 (2.0)	0.18	0.05
	No (ref)	116 (81.7)	22.5 (7.7)	21 (17; 27)						
Aphasia	Yes	9 (6.3)	24.8 (12.5)	21 (15; 34)	1.6 (2.8)	0.05	0.58			
	No (ref)	133 (93.7)	23.2 (7.9)	22 (17; 28)						

*SD* standard deviation, *Q1* first quartile, *Q3*, third quartile, *SE* standard error, *NS* not significant (p > 0.05) in multiple regression

^a^Variables with *p* values < 0.20 in the univariate analysis were entered in the multiple regression analysis. Multiple regression Model F_(3,138)_ = 10.953, *p* < 0.001, R^2^ = 19.2% (Adjusted R^2^ = 17.5%)

The FAS score was negatively correlated with all eight SF- 36 domain scores; that is, a higher level of fatigue was associated with worse quality of life (Table 4). Fatigue was moderately correlated, in descending order, with vitality (*r* = − 0.67), physical functioning (*r* = − 0.57), general health (*r* = − 0.57), mental health (*r* = − 0.57), social functioning (*r* = − 0.56), role–physical (*r* = − 0.53), and role–emotional (*r* = − 0.47). Fatigue was weakly correlated with bodily pain (*r* = − 0.38). The FAS score was moderately correlated (*r* = − 0.48) with the inability to return to pre-stroke life and activities; that is, people who were partly or fully unable to return to pre-stroke activities had higher FAS scores than those who were able to return to pre-stroke activities.

**Table 4:** Correlations between FAS scores and SF- 36, and return to activity, respectively

SF- 36 domains (*n* = 123 to 142)	Response categories	N (%)	Mean FAS score (SD)	Spearman’s rho (95% CI)
Physical functioning	N/A	134	–-	− 0.57 (− 0.68; − 0.44)
Role–physical	N/A	123	–-	− 0.53 (− 0.65; − 0.39)
Bodily pain	N/A	132	–-	− 0.38 (− 0.52; − 0.22)
General health	N/A	129	–-	− 0.57 (− 0.68; − 0.44)
Vitality	N/A	131	–-	− 0.67 (− 0.76; − 0.56)
Social functioning	N/A	132	–-	− 0.56 (− 0.67; − 0.42)
Role–emotional	N/A	125	–-	− 0.47 (− 0.60; − 0.32)
Mental health	N/A	131	–-	− 0.57 (− 0.67; − 0.43)
**Been able to return to prestroke life and activities (*n* = 139)**	Yes	54 (38.8)	19.3 (6.0)	− 0.48 (− 0.33; − 0.60)
	Yes, but not as before	51 (36.7)	23.8 (6.3)	
	No	34 (24.5)	29.7 (9.8)	

*FAS* Fatigue Assessment Scale, *SF- 36* 36-Item Short Form Survey, *CI* confidence interval

## Discussion

The main findings of this study indicate that vertigo, chronic pulmonary disorders, and hemiparesis are associated with more severe PSF. Additionally, higher fatigue levels were correlated with poorer QoL across all domains measured by the SF- 36. These results emphasize the multifaceted nature of PSF and its profound impact on QoL.

It is well known that fatigue can be a bothersome and persistent problem for stroke survivors. Damage to certain areas, such as the basal ganglia, internal capsule, brainstem, or thalamus, has been associated with increased fatigue, but fatigue can occur in any stroke patient, regardless of the size, location, or severity of the stroke. This phenomenon can be explained by different mechanisms, such as increased energy demands, sleep disturbances, inflammation, hormonal changes, and damage to certain critical areas, such as the basal ganglia, internal capsule, brainstem, or thalamus [7].

In this study, the variables “hemiparesis” and “aphasia” may be viewed as indicators of stroke severity. A study performed by one of the authors in 2006 revealed that both the National Institutes of Health Stroke Scale and Modified Rankin Scale, measured one year after stroke, were associated with increased levels of fatigue. In fact, the Modified Rankin Scale was the only significant predictor in multivariate analysis in that study [22].

Vertigo, or dizziness, is a common complaint among older individuals and often occurs together with a reduced balance and a tendency to fall. Fatigue is common during episodes of vertigo, particularly in cases of benign paroxysmal positional vertigo [23]. Several factors may explain the co-occurrence of vertigo and fatigue. Postural control requires attentional cognitive processing. Fatigue can impact these mental functions, potentially leading to disturbed postural control and dizziness. Vertigo can also disrupt sleep patterns, potentially leading to fatigue [24]. Fatigue is highly prevalent among patients with chronic pulmonary diseases. It is considered the second most important symptom after dyspnea in patients with chronic obstructive pulmonary disease (COPD). In COPD, prevalence estimates of mild-to-severe fatigue range between 47 and 72% [25].

Several other factors have been associated with fatigue in other studies. For example, there appears to be an association between cognitive decline and fatigue. In one study on stroke patients, fatigue was associated with lower scores on the Montreal Cognitive Assessment (MoCA) scale. There could be shared underlying mechanisms, as both fatigue and cognitive impairment are associated with factors such as sleep deprivation, stress, and certain medical conditions [26]. In our study, few responders were affected by cognitive impairment, psychiatric disorders and aphasia, which reduced the statistical power in the analyses. Thus, we cannot exclude that significant associations between these variables and PSF would have been found if the sample had been larger. Additionally, many studies have found an association between psychiatric disorders, notably depression, and fatigue. It is often one of the most prominent presenting symptoms of depression and can persist even after other depressive symptoms have improved with treatment [27]. It may also be possible that fatigue could increase symptoms of depression and negatively impact life [28].

Since many stroke patients are older and carry several diseases, it is often difficult to find a single cause of fatigue in individual cases. In one study, multiple comorbidities were identified as one of the strongest predictors of higher fatigue levels in stroke patients [29]. The additive effect of different comorbidities is unclear and cannot be reliably evaluated by the current study. Nor was this possible in the study by Kjeverud et al. [29], where multiple comorbidities were counted as 2 or more. It is possible that some comorbidities may have a potentiating effect on each other, but this is currently speculative. There is a lack of deeper knowledge about whether different diseases potentiate each other with respect to fatigue.

PSF affects all parts of the life of stroke survivors, including their physical functioning, mental well-being, and social relationships, and explains why many stroke survivors consider PSF to be among their worst stroke symptoms [7]. PSF often interferes with a person’s ability to engage in everyday activities and maintain independence. Specifically, it can make basic activities of daily living more challenging and energy consuming. Complex activities that require planning, multitasking, and problem solving become particularly difficult. In this context, it is unsurprising that the present study and others reported that PSF impairs QoL [30].

We found moderately strong correlations between PSF and seven of the eight domains of the SF- 36 and a weak correlation with one domain (bodily pain). We observed the strongest correlation for the vitality domain, which is consistent with the findings of two other studies [31, 32]. The vitality domain provides important information about a person’s overall health-related quality of life, particularly their energy levels and feelings of fatigue, which can impact daily functioning [33]. Vitality is operationalized in SF- 36 in terms of feelings of; pep, energy, worn out and tiredness, which are closely related to the operationalization of fatigue in FAS.

### Strengths and limitations

A limitation of this study was its cross-sectional design, which limits the ability to draw conclusions on causality. For example, factors measured by the SF- 36, such as poor physical functioning and mental health, could either result from fatigue or, conversely, contribute to its severity. This bidirectional relationship cannot be fully explored in a cross-sectional framework. Unfortunately, this study has a number of non-responders which could potentially have affected the results. This is particularly true for patients with cognitive impairment or aphasia, where a lower proportion participated in the study. It is also possible that patients with severe fatigue were less likely to respond to the questionnaire. This should be kept in mind when interpreting the results.

Another limitation was the absence of potentially relevant predictors, such as stroke severity, which were not available in the dataset. Furthermore, only quantitative data were collected. Supplementary qualitative interviews could conceivably have contributed to a more in-depth understanding of how individuals living with PSF experience their life with fatigue.

A limitation of patient record data is that while diagnoses may be convincingly reported, comorbidities and symptoms might be inconsistently documented, as data were collected for clinical rather than research purposes. Variability in practitioner reporting and coding practices should also be considered when interpreting the findings. Symptoms of stroke can also change over time, primarily because they often have a regressive course. This can lead to a loss of precision in the significance of stroke symptoms.

## Conclusion

The presence of vertigo, chronic pulmonary disorders, and hemiparesis were significant predictors of more severe PSF. Additionally, higher levels of fatigue were associated with a worse QoL across all domains. These findings suggest that PSF is a complex, multifaceted phenomenon that appears to be associated with certain comorbidities and reduced QoL. The results underscore the need for further research into the underlying causes and effective management strategies for PSF to better support stroke survivors in their recovery and rehabilitation.

## Data Availability

The datasets used and/or analyzed during the current study are available from the corresponding author on reasonable request.
